# An AutomationML Based Ontology for Sensor Fusion in Industrial Plants

**DOI:** 10.3390/s19061311

**Published:** 2019-03-15

**Authors:** Eder Mateus Nunes Gonçalves, Alvaro Freitas, Silvia Botelho

**Affiliations:** 1 Center for Computational Sciences, Universidade Federal do Rio Grande—FURG, Rio Grande, RS 96203-900, Brazil; silviacb@furg.br; 2 Instituto Federal Sul-Riograndense, Pelotas, RS 96203-900, Brazil; alvarofreitas@pelotas.ifsul.edu.br

**Keywords:** sensor fusion, AutomationML, ontology

## Abstract

AutomationML (AML) can be seen as a partial knowledge-based solution for manufacturing and automation domains since it permits integrating different engineering data format, and also contains information about physical and logical structures of production systems, using basic concepts as resources, process, and products, in semantic structures. However, it is not a complete knowledge-based solution because it does not have mechanisms for querying and reasoning procedures, which are basic functions for semantic inferences. Additionally, AutomationML does not deal with aspects of sensor fusion naturally. In this sense, we propose an ontology to describe those sensors’ fusion elements, including procedures for runtime processing, and also elements that can turn AutomationML into a complete knowledge-based solution. The approach was applied in a case study with two different industrial processes with some sensors under fusion. The results obtained demonstrate that the ontology allows describing sensors that are under fusion and deal with the occurrence of data divergence. In a broader view, the results show how to apply AutomationML description for runtime processing of data generated from different sensors of a manufacturing system using an ontology to complement the AML description, where AutomationML concentrates knowledge about a specific production system and the ontology describes a general and reusable knowledge about sensor fusion.

## 1. Introduction

Industrie 4.0 constraints require that information processing has to be enhanced towards a semantically integrated approach, which allows data analysis on big data coming both from product and production system life-cycle processes. In production system engineering, the current focus on data processing has to be moved on to information processing of semantically enriched data [1].

Knowledge-based approaches are able to deal with the data heterogeneity aspects of engineering of production systems. Moreover, they enable advanced capabilities, since they support: (i) the explicit representation of knowledge in a domain of interest and (ii) the exploitation of such knowledge through appropriate reasoning mechanisms in order to provide high-level problem-solving performance [1].

Originally, AutomationML (AML) is an open, XML-based data exchange format developed for supporting the exchange of diverse engineering data within the engineering process in manufacturing systems, including partially features of a semantically integrated approach [2]. AutomationML uses a representation of semantic mapping types that represent relations and dependencies between engineering models, which can be used to improve the machine-understandable modeling of the dependencies, enabling the automation of engineering process [3].

To enable the engineering and production processes for flexible production systems, integrated information processing intends to ensure the lossless exchange and correct (meaningful) application of engineering and runtime information of a production system to gain additional value and/or to avoid current limitations of production system engineering and use. However, AutomationML needs some complementary approach to include querying and reasoning methods in the sense of a more complete knowledge-based solution. An ontology can support data analysis across the discipline/tool boundaries in that process [2].

Additionally, in the context of integrated information processing on AutomationML, the aspects of multisensor fusion and integration are missing. Multisensor fusion and integration generate a synergistic combination of sensory data from multiple sources to provide more reliable and accurate information. Systems including multisensor fusion and integration have properties such as redundancy, complementarity, timeliness, and cost of information [4]. It can reduce overall uncertainty and serve to improve the accuracy with the features perceived from the system. Multiple sensors providing redundant information increase the reliability in case of failure or even errors [5]. Complementary information using multiple sensors allows inferring information from the environment that is impossible to perceive using information from individual sensors separately. Timely information has more accuracy since it is possible to perceive data from sensors with different speeds of operation [6]. Currently, AutomationML presents structures able to import data from different sources of sensors, including data from manufacturers of different brands, and even data from another AutomationML specification [7]. However, it is needed an external support to manipulate data from multisensor fusion and integration.

Using ontologies as a framework to reuse vocabularies, concepts and instances is a common practice, even when dealing with sensors.

On the other hand, some proposals using ontologies for the description and treatment of industrial information systems arise in the literature. A hydrological sensor web ontology based on Semantic Sensor Network (SSN) [8] ontology was proposed to describe heterogeneous hydrological sensor web resources by importing the time and space ontology, instantiating the hydrological classes, and establishing reasoning rules. This new ontology provides a new perspective on managing and responding to natural disasters, as well as reliable and efficient information to decision-making processes [9].

Ontology is also used as a context model for facilitating context sharing between heterogeneous devices and network optimizations, in this case exploiting context awareness in Wireless Sensor Networks [10]. The motivation is to provide a context model for modeling different situations and conditions of an Internet of Thing system with the aim of optimizing the underlying network using contextualized information.

Semantic Sensor Web is an area linking the elements from Semantic Web [11] technologies with sensor networks, and has among its main features the use of ontologies. One of its key challenges is to provide mechanisms to integrate and exchange knowledge from heterogeneous sources, which can be done using procedures for ontology alignment. These procedures can use fuzzy logic techniques to combine similarity measures between entities of different ontologies [12].

Another solution similar to ours also uses SSN as a base for a generic ontology used in the design and implementation of a sensor-based maintenance process. Its generic property permits using it to support the creation and management of other kinds of a sensor-based industrial process such as the continuous control of a production system [13]. Like our ontology, this one uses the knowledge obtained during the design process to aid in the maintenance design, especially about the sensors needed for monitoring that kind of tasks. However, all of this knowledge is integrated into the same ontology.

However, none of these solutions are able to reuse the whole knowledge available of a specific production system such as those described in an AutomationML file, which is able to integrate generic demands and descriptions of production system with data from specific types of equipment and devices.

The literature presents some recent research about the combination of AutomationML and ontologies, which can be considered as another framing of this work.

For fostering fast prototyping and reuse of robotic software, it has proposed a model-driven approach that uses AutomationML as modeling framework and ontological reasoning as inference framework for constructing robotic application using Robot Operating Systems (ROS) [14]. This approach is able to classify and model different robotic components with AutomationML, compose them together to a production system, and process these models semantically by utilizing Semantic Web technologies and ontological reasoning [15].

The combination AutomationML and ontology can also be used to validate plant models. The use of reasoning and querying for some validation features is possible along a CAEX plant engineering process using Semantic Web procedures [16]. Another possibility is to use ontology for matching and mapping methods in the context of data modeling for production planning, combining domain dependent knowledge from a Manufacturing Execution System (MES) with an AutomatiomML description [17].

The Ontology Web Language (OWL) can also be represented in the AutomationML and Open Platform Communications Unifying Architecture (OPC UA), enabling the collection and usage of data through the whole life cycle in OWL, and also extending the possibility for knowledge-based services such as self-configuration, self-optimization or self-diagnosis. However, this approach does not permit that OWL full ontologies be represented in the OPC UA information model. In addition, Semantic Web Rules Language (SWRL) rules cannot also be represented, but, by representing them in a string array, they are accessible [18].

The AutomationML Ontology (AMLO) has been proposed following best practices for ontology engineering, covering the entire CAEX standard, and not just portions relevant for a use case, and is open for the community by following latest guidelines on resource sharing and publishing [19].

When analyzing works under the perspective of a combination of AutomationML and ontologies, any of these approaches are unable to integrate general conceptions of a production system, such as arrangements of fusion sensors on the field, with specific engineering data, such as those in an AutomationML description, and inferring those arrangements automatically.

The main research question addressed in this paper is how to take advantage of AutomationML as a complete knowledge-based solution for the engineering, operation and maintenance of a production system? What is needed to complement AutomationML as a complete knowledge-based solution? How does one integrate knowledge of different forms of combinations of sensory data in AML descriptions?

With this background information, we propose in this paper an ontology which is complementary with AutomationML targeting a complete knowledge-based approach. The goal is to provide AutomationML with structures and procedures that make it possible to represent the different techniques of fusion of sensors and share this information with the information systems that manipulate runtime data of the plant, namely, control and supervision systems. Herewith, it is possible to take advantage of the whole topological information contained in the AutomationML description for monitor, supervisory and control tasks, while it is possible to execute querying and reasoning methods on this information. Even that AutomationML was previously conceived to be a storage format for engineering data, it has a potential for a full semantic solution since it is able to represent data of different disciplines that compose a production system, which by itself is a powerful knowledge source for any information system. The proposed ontology was validated in a case study based on two different industrial processes with some sensors under fusion. The approach is able to describe all existent fusion in the plant pointing out the occurrence of data divergence. Thus, the developed ontology can be considered a compliment for AutomationML able to turn it in a full knowledge-based solution. The main contribution of the paper is an approach to complement the specific knowledge of a production system described in AutomationML with general conceptualizations about sensor fusion in an ontology, which in its turn can be reused on another systems. This approach reduces the time and work necessary to integrate new sensors in the field, once the AutomationML description acknowledges them straightaway and the Ontology can identify if any sensor will integrate some fusion arrangement.

The paper is organized in the following way. The next section describes the whole theoretical background needed for the ontology conception and implementation. Section 3 presents the ontology description. Section 4 presents a case study applying the developed ontology and, finally, Section 5 describes a final synthesis of this work and some reflections about future works.

## 2. Background

Industrie 4.0 envisions the dynamic integration of components within the production systems runtime looking for a sufficient resource-related flexibility. To make integration possible, two information processing features are required. First, the newly integrated production system component has to provide information about (i) its capabilities, (ii) its access paths, and (iii) its control-related features. Second, the overall control system of the production system must be able to reason about the component information and also integrate it at runtime within the processes [1].

On the other hand, Industrie 4.0 also demands product-related flexibility since it assumes that each product should know the required material and processes for its creation. In this context, the information will be used for the identification of the manufacturing resources and to parameterize the processes executed by applying the related values within the control systems.

A common process description based on the *product-process-resource* concept would be desirable. This description would permit expressing the concepts and relationships in a semantically well-defined and comparable way, about: (i) the needs for capabilities of production system components and devices; (ii) the component/device use conditions (access path and control); and (iii) product-related processing requirements [1]. In summary, according to this view, the intention of any production system is to execute production processes using production resources in order to create products [20].

AutomationML is a solution for integrating data of different disciplines needed to conceive production systems, especially recommended when using Cyber-Physical Systems (CPS) paradigm [21], for example. It was conceived as an XML based data format permitting the exchange of plant engineering information [7].

The AutomationML approach is based on a three-view-concept for interlinked engineering data: resources, process, and products. *Resource* is an entity in production which executes processes and handles products. It can be hardware components or even software: robots, conveyors, machines; SCADA systems. A *product* depicts a produced good. It is processed by resources and can be built up hierarchically and described by an assembly tree. Finally, a *process* represents a production process including sub-process, process parameters, and the process chain. In addition, processes modify products.

AutomationML includes information about system topology, geometry, kinematics, and control behavior and allows including links to detailed engineering models representing such information, like COLLADA or PLCopen files [20].

AutomationML follows the object oriented paradigm and allows modelling of physical and logical components as data objects encapsulating different aspects. Thus, an object may consist of different sub-objects, and also be part of a larger composition or aggregation. Typical objects may be information on topology, geometry, kinematics and logic, whereas logic comprises sequencing, behavior and control. The core of AML is the top-level data format CAEX, which is used to interconnect the different data formats. Therefore, AML has inherent distributed document architecture [22].

AutomationML can store and exchange information from [22]:

- **Plant topology information**: a hierarchical structure of individual plant objects, which are represented by individual data objects. The plant topology acts as the top-level data structure and it is stored by means of the CAEX data format.
- **Geometry and kinematics information**: The geometry of an object comprises its geometrical representation, whereas the kinematics information describes the physical connections of 3D solids and dependencies among objects. Both types of information are manipulated using the file format COLLADA.
- **Logic Information**: It describes sequences of actions and the behaviour of objects including I/O connections and logical variables. Sequences are manipulated based on PLCOpen XML documents, and variables or signals are published as CAEX ExternalInterfaces.
- **Reference and relation information**: References are links from CAEX objects to externally store information, whereas relations are associations between CAEX objects.

A complex production system generates an immense volume of heterogeneous data, which must be provided in real time for the all control and supervisory systems. For working properly, it must guarantee the correctness of data provided within real-time requirements for those systems.

Working as a network, scalability, and redundancy can be more naturally approached when dealing with sensors data and for generating information for upper systems with reliability and integrity. However, those properties can only be achieved once strategies for data treatment are applied.

The literature presents some different nomenclatures to refer about using a set of sensors in an integrated way. The terms “data fusion”, ”sensor fusion”, “multi-sensor integration” have been used to refer to a variety of techniques, technologies, systems, and applications that employ and/or combine data derived from multiple information sources. More strictly, sensor fusion is about the combination of data from multiple sources to provide enhanced information quality and availability over that which is available from any individual source alone [23].

If we consider production systems with properties like flexibility, reconfigurability, and especially, with plug-and-play capabilities, traditional techniques for integration of multi-sensor are not a good solution once they are normally based on static topological structures. It would be interesting in systems based on CPS concepts, for example, that, when replacing a device, the whole system would recognize the new device and automatically insert it into the compounded sensors’ arrangement.

A common situation when reading data from multiple sources is the presence of different readings for the same variable since we can have multiple sensors responsible for the same phenomena. In this context, we consider that the system presents *data divergence* between sensors responsible for the same variable.

However, AutomationML is not able to deal with run-time divergent information, which would be interesting for maintenance procedures, for example. The engineering knowledge available in AutomationML could be applied to ensure the correct integration of new devices within replacement strategies. Besides that, a reasoning and querying mechanism would permit some level of guarantee for new system components fulfilling the needs of the application, even if the new component is not an exact copy of the component being replaced.

To incorporate those features, we propose an ontology for complementing AutomationML that enables it to make queries and inferences about the whole knowledge of the production system including runtime processing from plant data. Besides that, the ontology must present elements to represent arrangements of sensor fusions indicating the occurrence of divergence between data when they happen. Those features are relevant for the whole set of control and supervisory system that can act correctly based on a more reliable data set.

A sensor ontology for manufacturing must include [24]:

- detailed standard knowledge representation of sensor physical dimensions, weight, resolution, associated system performance, and operating conditions;
- representation of system capabilities, to categorize the functions that individual and groups of sensors can perform;
- representation of sensors embedding in sensor networks and the manufacturing environment.

Besides those features, we include the representation of arrangements for sensor fusion indicating when divergence of data between the sensors of a specific arrangement occurs.

Unfortunately, reusing ontologies on the automation and manufacturing domains is not a usual practice. This is exemplified by the issues we found about different approaches for modularity, absence of public licenses and documentation, and, mainly, due to heterogeneous application fields (e.g., industry, building, grid automation) and due often to strong dependencies between vendor-specific hardware devices and tooling [25]. Besides that, since a strong requirement for us was to build a complementary ontology for AutomationML, we decided to propose a new ontology.

The ontology described in this work was developed using the methodology named *Methontology* [26], which was chosen because it presents a formal conception for the whole life-cycle of development for ontologies: (i) requirements specification; (ii) conceptual modeling; (iii) formalization; (iv) implementation; (v) integration; (vi) assessment; and (vii) documentation. A special feature required is the capability of integrating with another ontologies or data description, which was important in the sense of a complementary AutomationML.

The next section describes the ontology developed to complement AutomationML capabilities as a full knowledge-based solution.

## 3. An Ontology for Complementing AutomationML

Once we select *Methontology* as the methodology to build our ontology, it was necessary to search for a knowledge source to provide concepts and relations between the manufacturing domain elements, in order to define a taxonomy. Besides that, it was also defined the type of user for the ontology.

**Figure 1:**
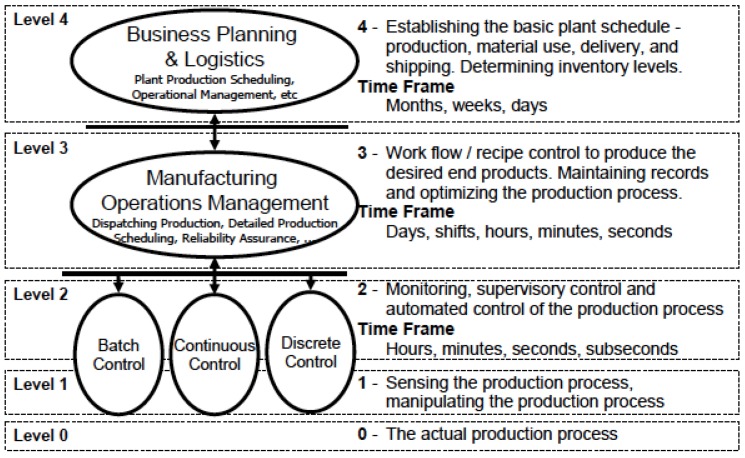
The automation pyramid [27].

Regarding the users, the classical picture of the ISA-95 automation pyramid, replicated in Figure 1, is taken as Ref. [27]. Our ontology is intended to be manipulated by operators working on levels 0, 1, 2, and 3, namely, field technicians and engineers.

**Figure 2:**
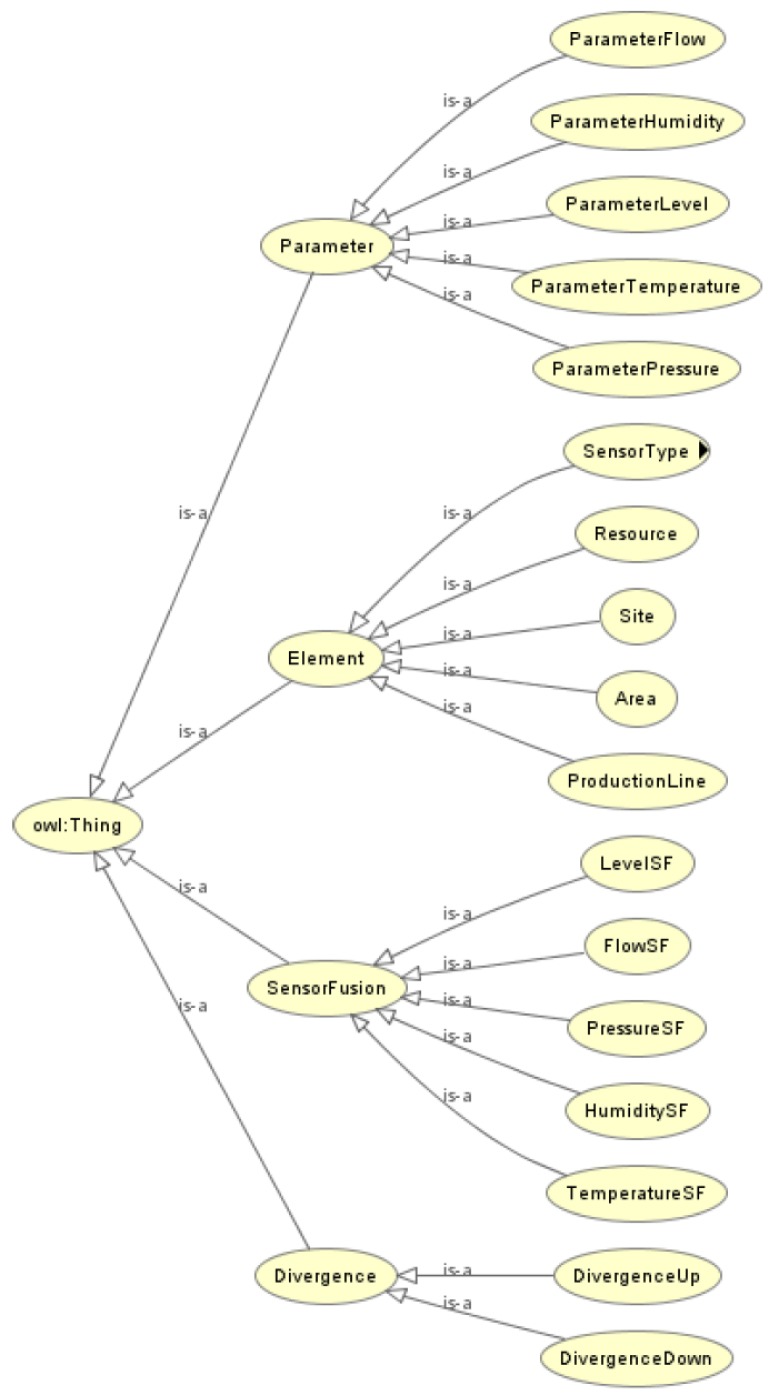
A partial description of the proposed ontology.

The taxonomy was designed based on the AML descriptions, using International Electrotechnical Commission (IEC) standards to formalize concepts [28] and its relations. Following the taxonomy described in Figure 2, it has a main root named generically *Thing*, which is followed by four main distinct modules: (i) *Element* where the physical components of a plant are described; (ii) *SensorFusion*, where components present in sensor fusion are listed; (iii) *Parameters*: a list of parameters for each measured phenomena (pressure, flow, level, temperature, ...) for each resource; and (iv) *Divergence* for divergence detection under fusion measures.

**Figure 3:**
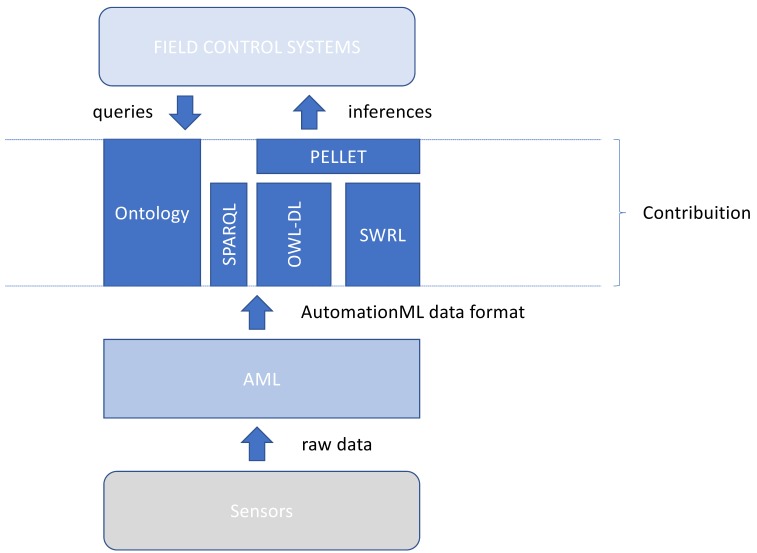
Ontology description.

Figure 3 describes the theoretical conception of our knowledge-based solution, integrating base components of a full semantic solution. According to this figure, the proposed ontology is inserted such as middleware between sensors in the plant and the control system that will manipulate it. The AML must be loaded with runtime data from the field. This composed structure, AML + data, is instantiated inside the ontology through a parser procedure, which enables all kind of semantic procedures, such as queries and reasoning actions. In Section 4, we present a possible implementation solution for this model using manufacturing standard technologies.

**Figure 4:**
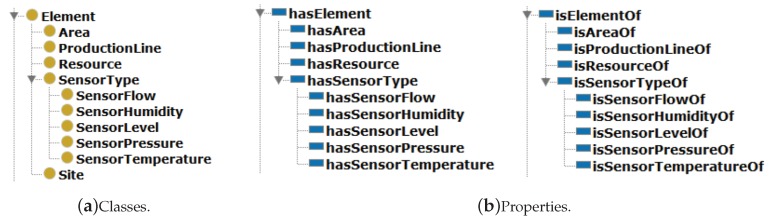
Classes (yellow) and Properties (blue) of module *Element*.

It is not possible to produce a legible figure with a full taxonomy description since there is no space for it in a single page. A partial description of our ontology, including all modules and some classes, is presented in Figure 2. However, each module, class, and properties are detailed in the next subsections. In fact, for a complete description, it lacks the subclass of the *SensorType* class. However, those missing data can be checked at Figure 4.

### 3.1. The Element Module

**Figure 5:**
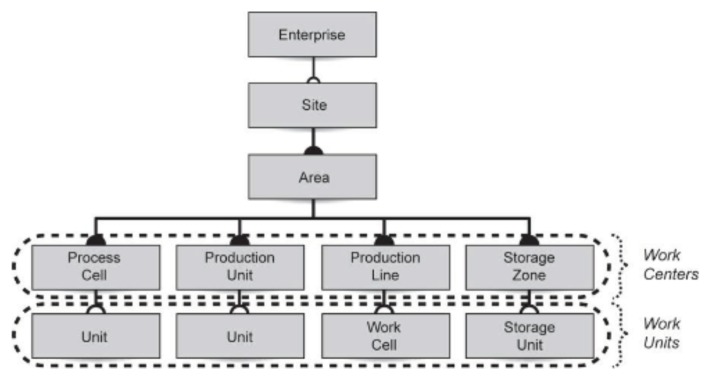
The physical component conception inside the proposed ontology [28].

Figure 5 describes the physical elements that were conceived in a module named *Element*, which is based on IEC 62264-1:2013, defined as an hierarchical taxonomy of physical structures, production lines and resources, and the last level contains the different kind of sensors.

The module *Element* is composed of the following classes:

- Class *SITE*: a physical, geographic or logical grouping that is composed of a physical area, production lines, process cells and production units.
- Class *AREA*: a physical, geographic or logical grouping determined by the *SITE*. It can contain process cells, production units, production lines, and storage zones. An area is related to just one site. Nevertheless, an area can be composed of many production lines.
- Class *PRODUCTIONLINE*: main activities of processes and/or assembly are allocated to a production line. In the hierarchical taxonomy proposed by IEC 62264-1, production lines and work cells are the lowest levels for equipment. However, work cells are normally applied when there are demands for routing inside of a production line.
- Class *RESOURCE*: this class was included as a basic type of AML, once it is not part of the IEC standard. It is used to describe any kind of production resource, and in the ontology proposed in this paper, resources are equipment like tanks, pipes, where sensors under fusion are directly installed. A resource has a relationship with only one production line.
- Class *SENSORTYPE*: it was designed to represent sensors and is also an AML abstract type, as the class *RESOURCE*. There are specific subclasses for each type of sensors that were declared as disjunct. One sensor has a relationship with one resource, but, once there are different types of sensors, there is a specific *domain* for each type, but with the same *range*, defined by the class *RESOURCE*.

Figure 4 shows the different classes and properties inside the *Element* module.

### 3.2. The Sensor Fusion Module

*SensorFusion* module is composed of a set of classes applied to identify individuals (sites, areas, production lines, resources and sensors) present in a sensors’ fusion. All classes at this module are high level classes, and the individuals at this class are logical instances. Individuals at high level classes are inserted by SWRL rules [29], once it is necessary to activate the inference engine to detect the condition associated with the sensor fusion, which includes at least one resource and two sensors of the same type.

**Figure 6:**
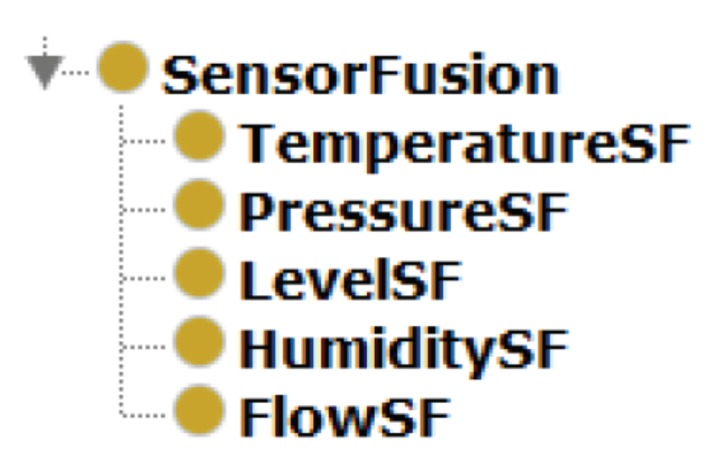
Sensor fusion module.

The subclasses at this module are declared as disjunctive to avoid that a sensor belongs to two or more different fusions. *SensorFusion* module is described in Figure 6.

The first SWRL rule, from now on named rule(1), defines a fusion when a resource is composed of at least two sensors of the same type. In case of level sensors, the SWRL rule is:

Resource(?r),SensorLevel(?sl1),SensorLevel(?sl2),hasSensorLevel(?r,?sl1),

hasSensorLevel(?r,?sl2),DifferentFrom(?sl1,?sl2)→SensorFusion(?r),

LevelSF(?sl1),LevelSF(?sl2),

where *r* is a resource, and sl1 and sl2 are level sensors.

The second SWRL rule, named rule(2), is necessary to indicate upper levels in the ontology if fusion exists below that level. For example, the following rule indicates that, if a resource has sensors in fusion and it belongs to a production line; consequently, this production line has elements in fusion:

Resource(?r),SensorFusion(?r),ProductionLine(?p1),

hasresource(?p1,?r)→SensorFusion(?p1),

where *r* is a resource and p1 is a production line.

The SWRL rule(3) defines which areas has sensor fusion, and inherit the definitions of both previous rules. In this case, if an area has a production line that has sensor fusion; consequently, this area has elements in fusion. The rule(3) is:

Area(?a),ProductionLine(?pl),SensorFusion(?pl),

hasProductionLine(?a,?pl)→SensorFusion(?a),

where *a* is an area and p1 is a production line.

The last rule about physical elements, rule(4), and the highest level in the ontology structure refers to sites. As the previous rules, it inherits the definitions and is defined in the following way: if a site has an area and this area has sensor fusion; consequently, this site has elements in fusion:

Site(?s),Area(?a),SensorFusion(?a),hasArea(?s,?a)→

SensorFusion(?s),

where *s* is a site and *a* is a area.

### 3.3. The Parameters Module

**Figure 7:**
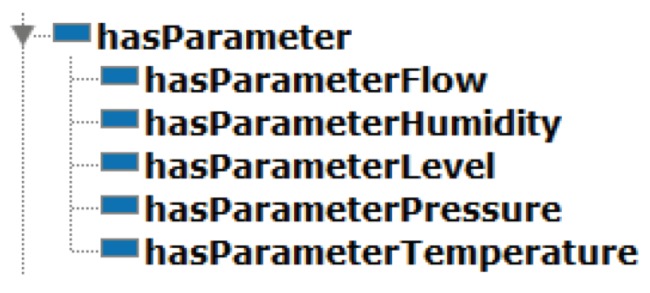
*Element* module inverse properties.

The *Parameters* module is able to represent parameters for each resource in a sensor fusion, including each sensor type described in the ontology. Individuals in this class are parameters with two data properties used to specify the minimal and maximal values for each resource for each sensor type that belongs to a fusion. Object properties of the *Parameter* module are described in Figure 7.

**Figure 8:**
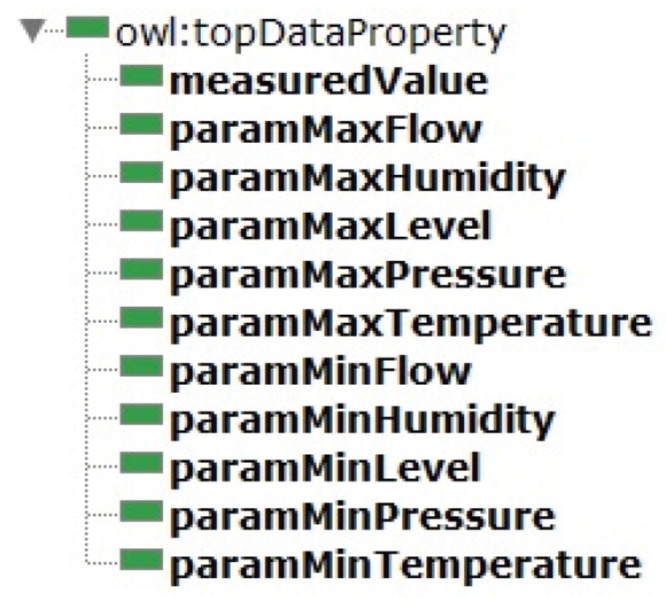
Data properties for parameter types.

*Parameters module* properties are specified for each sensor type, and all of them have the same Domain, the *Resource* class, but with a specific range for each sensor type. The data properties for parameters type are described in Figure 8.

### 3.4. The Divergence Module

**Figure 9:**
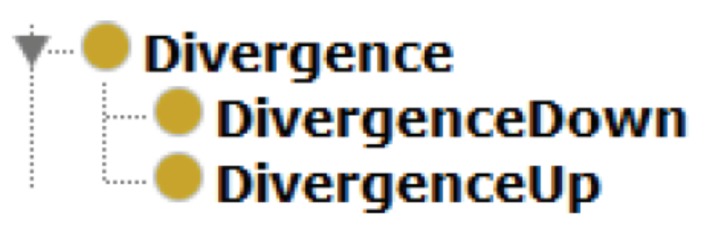
The *Divergence* module.

The *Divergence* module is composed of *DIVERGENCEDOWN* and *DIVERGENCEUP* classes. They are also high level classes, in a way that *DIVERGENCEDOWN* indicates occurrences of divergences below the minimal parameter defined for a specific phenomena in a specific resource, and *DIVERGENCEUP* when the divergence occurrence is above the maximal parameter of a specific phenomena in a specific resource. The constraints of both classes are specified by SWRL rules. The module *Divergence* is described in Figure 9.

Detecting divergence above the defined limit is possible applying this SWRL rule, i.e., rule(5):

Resource(?r),SensorFusion(?r),SensorLevel(?s),hasSensorLevel(?r,?s),

measuredValue(?s,?mv),ParameterLevel(levelt1),hasParameterLevel(?r,levelt1),

paramMaxLevel(levelt1,?pmax),swrlb:greaterThan(?mv,?pmax)→DivergenceUp(?s),

where *r* is a resource, *s* is a level sensor, mv is a level parameter, levelt1 is the maximum value defined for a level parameter and pmax is the maximum value measured. This rule uses a SWRL function, swrlb:greaterThan, from a specific SWRL mathematical library, which returns TRUE if the function premise value is greater than the function conclusion value.

The rule(6) uses the same principle of rule(5), but for detecting divergence below of the defined limit and applying the swrlb:lessThan SWRL function:

Resource(?r),SensorFusion(?r),SensorLevel(?s),hasSensorLevel(?r,?s),

measuredValue(?s,?mv),ParameterLevel(levelt1),hasParameterLevel(?r,levelt1),

paramMaxLevel(levelt1,?pmax),swrlb:lessThan(?mv,?pmax)→DivergenceDown(?s).

## 4. A Case Study

A case study was deployed using two different industrial processes in a fusion context. Both processes are conceived to heat a fluid, which can be done in an isolated or integrated way. In this sense, the processes have sensors for level, temperature, and pressure, besides some actuators to manipulate the fluid between different tanks. The difference between those processes is that the first one has more sensors than the second one. It was performed in SMAR’s Instructional Didactic Plant that is designed to demonstrate the operation of several control loops using the same equipment and configuration tools developed for industrial control applications [30].

The goal was to validate the proposed ontology integrated with topological information from an AutomationML description, demonstrating that it is able to identify which sensors are in fusion and where they are located in a plant. In this case, only level sensors were employed, since the procedure is the same to other types of sensors. Each process had the following description:

- **Process1**: Site: *process1* / Area: *building1* / Production Line: *smar1* / Resource: *tank1*/ *tank1* has three level sensors: sl1, sl2 and sl3 / *tank1* has a level parameter: *levelt1*;
- **ProcessA**: Site: *processA* / Area: *buildingA* / Production Line: *smarA* / Resource: *tankA*/ *tankA* has two level sensors: slA and slB / *tankA* has a level parameter: *leveltA*.

**Figure 10:**
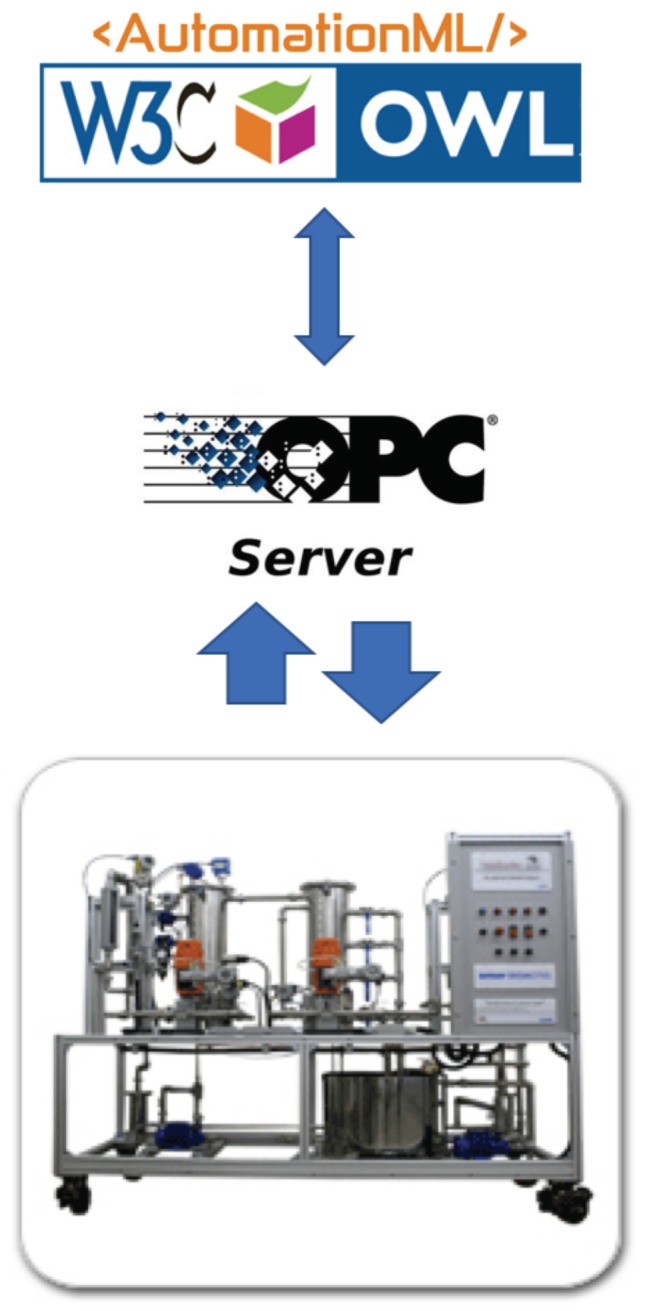
The case study components.

The runtime data from the processes is instantiated in the *AutomationML* description of the process evaluated, which generates an XML file. Using this description, the integrity about the plant structure and data generated is guaranteed. Currently, this process is executed by an external software coded in C language. However, since there is not any inference engine for AutomationML descriptions, an ontology OWL instance [31] is also necessary running in a Protégé environment [32]. Then, data from the plant with AutomationML description are sent to an OPC server, which is an OWL instance. Figure 10 describes the whole process including the plant, OPC server, AML, and OWL descriptions. In fact, Figure 10 is a specific implementation of Figure 3, since the plant and the OPC server execute the role of the *Sensors* block, and the *AML* block and the whole blocks of the *Ontology* are represented by the W3C and AutomationML logos.

**Figure 11:**
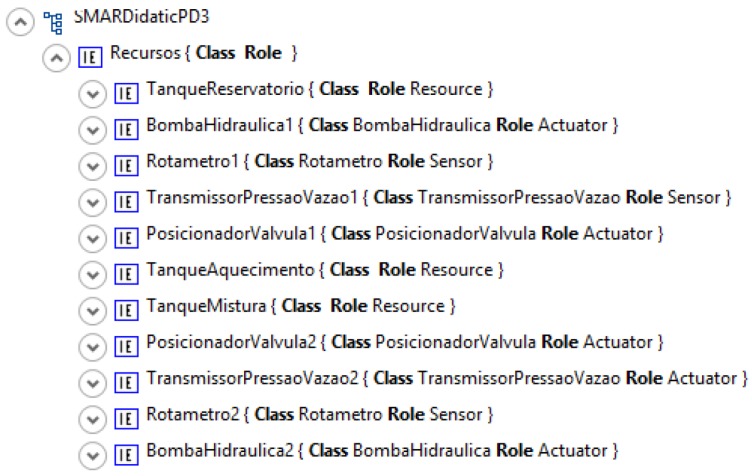
AML partial description.

An AML partial description is presented in Figure 11, where the plant resources and the related classes are defined.

Once the OWL and AML descriptions have loaded plant data from the OPC server, a set of tests to validate the structure of fusion sensors in the process has been carried out. In this sense, four different queries to the ontology were made: (i) which resources and sensors are in fusion; (ii) which line production has some fusion; (iii) which areas has some fusion; and (iv) which sites have some fusion.

**Figure 12:**
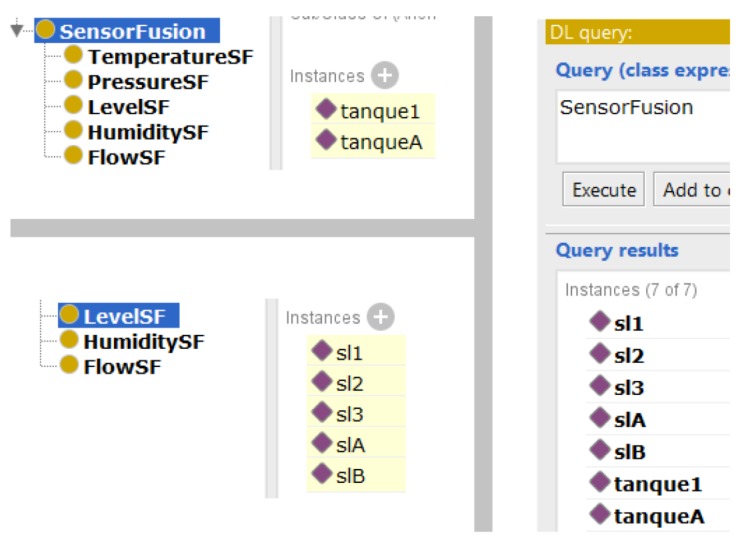
The first query: the upper left side shows the resources with fusion, and on the same side below shows the level sensors that are in fusion; on the right side, the *DL Query* including all the elements in fusion is shown.

The first query is a compound one, once it is necessary to characterize logical and physical conditions, i.e., which resources have sensor fusion and which sensors are in those fusions. Running the inference engine of the Protégé, the Pellet [33], the results in Figure 12 are returned.

The first query presented in Figure 12 was validated, since it has returned the correct aggregation, i.e., *tank1* with three sensors sl1, sl2 and sl3, and *tankA* with both level sensors, slA and slB.

When conceiving the scenarios for the case study, different quantities of sensors for each tank were included to verify if a query like this one could cater to minimal quantity requirement, i.e, a fusion with only two sensors, and above the minimum, when it is grouped with the resource, which also returned a correct answer. In addition, when a sensor in *tank1* was removed, and, in *tankA*, the resource was removed, Pellet did not indicate fusion for both cases.

**Figure 13:**
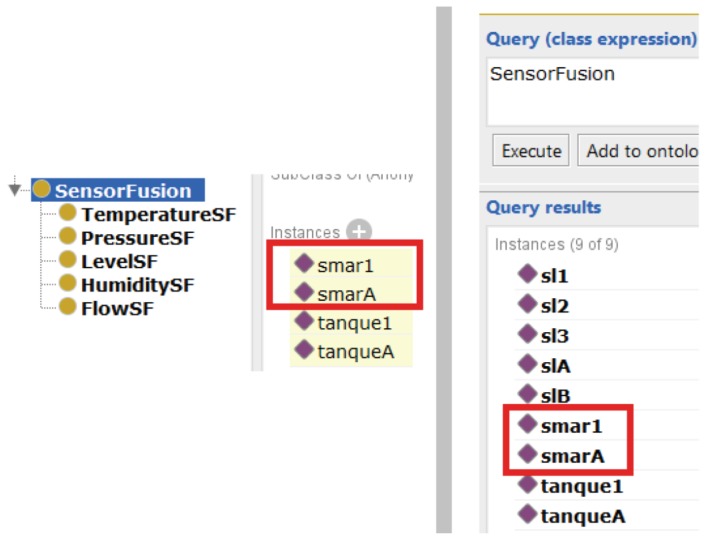
The left side shows the elements and, with a red square, the production lines with fusion. The right side shows the *DL Query* with all elements that are in fusion, including the production lines.

The second query to be evaluated is to infer which production lines have sensor fusion. This query is situated in the next level after sensors and resources. The production lines level is composed by those two elements. Figure 13 describes the results of this query, since tank1 was assigned as from production line smar1, and tankA was assigned as of the production line smarA. Since both tanks have sensor fusion; consequently, both production lines also have fusion.

The same procedure adopted in the first query was also applied for the second one, i.e, some components from the lower level were removed, which caused Pellet to not identify any fusion in any of the production lines. In these terms, the second query was considered correct.

**Figure 14:**
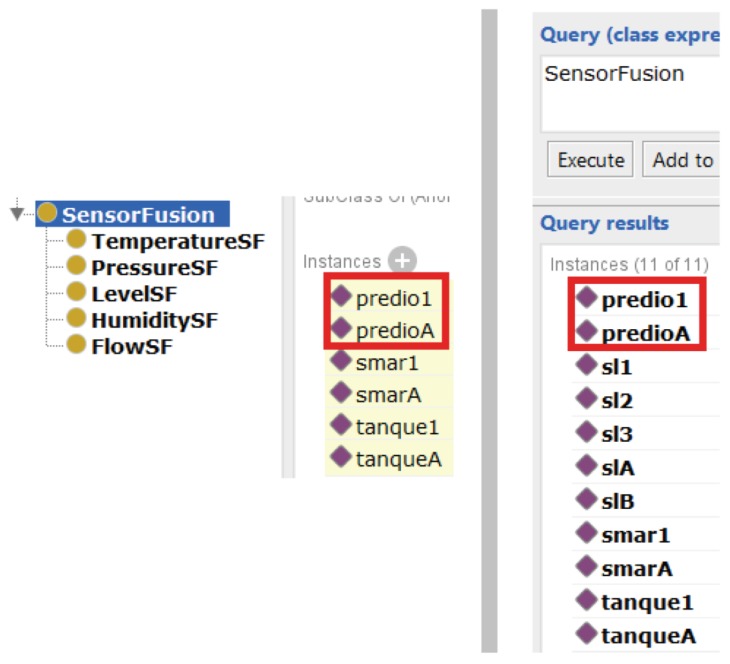
The left side of the figure shows resources, production lines and, inside of the red square, areas in fusion. The right side of the figure shows the *Description Logic (DL) Query* with all the elements that are in fusion, including areas.

The third query aims to validate rule(3), indicating sensor fusion under an area, as demonstrated by Figure 14. In the same way as previous ones, this query was able to identify the areas in fusion, since production line smar1 was assigned to builing1 and smarA was assigned to buildingA. In addition, the same procedure removing some elements of the fusion was performed and the Pellet did not identify any fusion, as it was supposed to do. Thus, the third query was considered also correct.

**Figure 15:**
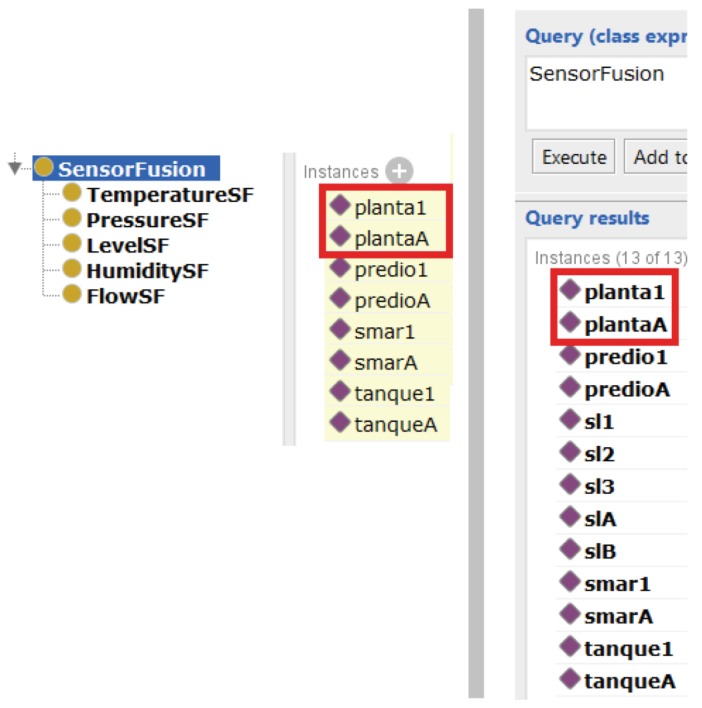
The left side shows resources, production lines, areas and, inside of the red square, sites with sensor fusion. The right side shows the *DL Query* with all the elements that are in the fusion, including sites.

The last query about the physical elements of the ontology was also successful. The results are presented in Figure 15. The inference returned that the area bulding1 was assigned to site process1 and the area buildingA was assigned to site processA, as expected. Since both areas have sensor fusion, the inference returned that both sites have elements in fusion. In addition, the same procedure removing individuals of fusions was performed, which immediately was identified by the Pellet indicating no more fusion.

Regarding the last rules, only data properties for parameter types considering continuous values were demonstrated. However, a specific way to handle binary signals may be described, where it would return FALSE in case of some divergence, or TRUE otherwise. This rule was not demonstrated because the SMAR didactic plant does not have any instrument with those properties.

This case study was carried out with a few sensors. However, extending this scenario with hundreds or even thousands of sensors, it is possible to distinguish two main components: (i) the knowledge base about the production system, considering its devices, structure and capabilities, which it is described by the AML description; (ii) and the queries and inference mechanisms, implemented in a general way, can be reused for different AML descriptions. In this sense, one can imagine the development of different oil refineries, which will produce different kinds of fuels. This scenario will be composed by production systems with thousands of tags associated with some Programmable Logic Controllers (PLC). Each refinery will be characterized by its process and capabilities, which will be featured by different AML descriptions. However, queries and inference mechanisms about the sensor fusion can be easily reused for both production systems. In addition, it is important to consider that even AutomationML has mechanisms to import knowledge from other AML descriptions, which can anticipate steps in the constitution of production system in different scales.

Another important aspect to be considered is the simplicity of SWRL rules and also of the ontology as a whole. This due to the fact that most of the knowledge necessary to represent fusion arrangements are already defined in the AutomationML description, which, in the process of a production system development, is a mandatory requirement in the sense of full semantic integration. According to rule (1), a fusion is identified if sensors are from the same type and also if they pertain to the same resource, and the resource is defined in the AML description. If properly structured, the AML enables the ontology to identify different types of fusion arrangement. However, if it is not the case, the additional specifications must be made in the conception of the resource inside the ontology description.

Finally, an important assumption about the presented approach is that we develop the ontology aiming to identify and indicate some kind of divergence for the monitoring and control systems, considering that those systems will handle this divergence. This assumption was made since each control system has specific requisites about data accuracy and reliability. In this sense, we understand that our case study attends all requirements for the approach validation.

## 5. Conclusions

AutomationML is a powerful industrial standard in order to integrate different engineering data formats used to design and implement complex production systems. However, it is not a complete solution since it lacks procedures for querying and reasoning tasks when proposed as a knowledge-based solution and also lacks basic structures to represent basic arrangements as fusion sensors, which is very common in this environment. In this paper, we proposed an ontology for complementing AutomationML fulfilling this gap. It is based on IEC standards complemented with some specific AML conceptions. The main contribution of the proposed approach is to integrate all of the knowledge available on an AML description, about physical and logical structures of production systems with general conceptualizations about fusion sensors. The ontology can be reused with other AML descriptions when dealing with fusion sensor arrangements in other systems. Besides that, it permits all kinds of semantic queries and reasoning including the knowledge available in the AutomationML and in the ontology itself. Most works in the literature use some kind of standard ontology for sensors in order to reuse general definitions, including specific aspects of each domain applied. However, they integrate all of the knowledge in only one extended ontology, becoming difficult to reuse them; once, in these cases, the ontology is tailored for specific domains, becoming almost an ad hoc solution.

The approach proposed was validated in a case study composed of two different industrial processes implemented in SMAR’s Instructional Didactic Plant. Those processes illustrate the most basic conception of sensor fusion, and also the most difficult situation to identify it since the arrangements have a minimal quantity of sensors for a fusion. The ontology is able to identify all sensor fusion presented in the plant, and where they are located, in runtime, as well is able to identify when a fusion is disassembled. In this sense, the ontology aggregates elements to an AutomatiomML description, becoming a complete knowledge-based solution.

It is intended as future work to incorporate a routine inside an OPC server for feeding the AutomationML description with data from the field. Currently, this procedure is executed by an external software coded in C. The resulting file of this process must be instantiated inside the ontology OWL description, which already is done using the temporary C routine. The new version to be developed integrates all routines for OWL and AML integration inside an OPC server. Besides that, we also intend to integrate AutomationML and this ontology to CPS reference architectures, as, for example [21,34], since it will naturally include some important requirements for this kind of solution as, for example, interoperability, reconfigurability, and flexibility, which can decrease the time necessary for engineering, development, maintenance, and operation of production systems, increasing industry efficiency and productivity.

## Abbreviations

The following abbreviations are used in this manuscript:

AML	AutomationML
AMLO	AutomationML Ontology
CPS	Cyber-Physical Systems
IEC	International Electrotechnical Commission
MES	Manufacturing Execution System
OWL	Ontology Web Language
XML	Extensible Markup Language
SWRL	Semantic Web Rule Language
OPC UA	Open Platform Communications Unified Architecture
SCADA	Supervisory Control and Data Acquisition
